# Dataset for the combined transcriptome assembly of *M. oleifera* and functional annotation

**DOI:** 10.1016/j.dib.2020.105416

**Published:** 2020-03-20

**Authors:** K. Mohamed Shafi, Adwait G. Joshi, Iyer Meenakshi, Shaik Naseer Pasha, K. Harini, Jarjapu Mahita, Radha Sivarajan Sajeevan, Snehal D. Karpe, Pritha Ghosh, Sathyanarayanan Nitish, A. Gandhimathi, Oommen K. Mathew, Subramanian Hari Prasanna, Manoharan Malini, Eshita Mutt, Mahantesha Naika, Nithin Ravooru, Rajas M. Rao, Prashant N. Shingate, Anshul Sukhwal, Margaret S. Sunitha, Atul K. Upadhyay, Rithvik S. Vinekar, Ramanathan Sowdhamini

**Affiliations:** a National Centre for Biological Sciences (TIFR), GKVK Campus, Bangalore 560065, Karnataka, India; b The University of Trans-Disciplinary Health Sciences & Technology (TDU), Yelahanka, Bangalore 560064, Karnataka, India; c Department of Biotechnology, Thapar Institute of Engineering and Technology, Patiala, Punjab 147004, India

**Keywords:** Transcriptome, Annotation, Orthology, Gene expression, Enrichment analysis, Metabolic pathway

## Abstract

In this paper, we present the data acquired during transcriptome analysis of the *plant* Moringa oleifera [1] from five different tissues (root, stem, leaf, flower and seed) by RNA sequencing. A total of 271 million reads were assembled with an N50 of 2094 bp. The combined transcriptome was assessed for transcript abundance across five tissues. The protein coding genes identified from the transcripts were annotated and used for orthology analysis. Further, enzymes involved in the biosynthesis of select medicinally important secondary metabolites, vitamins and ion transporters were identified and their expression levels across tissues were examined. The data generated by RNA sequencing has been deposited to NCBI public repository under the accession number PRJNA394193 (https://www.ncbi.nlm.nih.gov/bioproject/PRJNA394193).

Specifications table

**Table:** 

Subject area	Biology
More specific subject area	Plant biology; Bioinformatics
Type of data	Transcriptome and gene annotation data (Graphs, figures, tables)
How data was acquired	RNA sequencing
Data format	Raw, analysed
Experimental factors	RNA extraction, sequencing, de novo transcriptome assembly and annotation, data analysis
Experimental features	Total RNA extracted from five different tissues (leaf, root, stem, seed and flower) for sequencing.
Data source location	*Moringa oleifera* Bhagya variety (KDM-01), collected from University of Agricultural Sciences, Bangalore, India
Data accessibility	RNA-Seq data from this study have been submitted to the NCBI Sequence Read Archive – SRA at http://www.ncbi.nlm.nih.gov/Traces/sra/sra.cgi with accession numbers: SRX3011282 (Stem), SRX3011281 (Root), SRX3011280 (Pod/Seed), SRX3011278 (Leaf), SRX3011259 (Flower) Analyzed data files available at: http://caps.ncbs.res.in/download/ddat_dib/
Related research article	Naseer Pasha S, Shafi KM, Joshi AG, Meenakshi I, Harini K, Mahita J, Sajeevan RS, Karpe SD, Ghosh P, Nitish S, Gandhimathi A, Mathew OK, Hari Prasanna S, Malini M, Mutt E, Naika M, Ravooru N, Rao RM, Shingate PN, Sukhwal A, Sunitha MS, Upadhyay AK, Vinekar RS and Sowdhamini R. The transcriptome enables the identification of candidate genes behind medicinal value of Drumstick tree (*Moringa oleifera*). Genomics, https://doi.org/10.1016/j.ygeno.2019.04.014

## Value of the Data

• This data provides a transcriptome assembly of *M. oleifera* along with downstream analysis including relative abundance, orthology relationships and function assignment.
• A platform for identification of enzymes involved in biosynthesis of secondary metabolites, vitamins and ion-transporters with help of an improved bioinformatics pipeline.
• The data will allow the scientific community to carry out additional analysis for commercial production of the secondary metabolites.

## 1 Data

Data reported here contains a combined transcriptome assembly of five different tissues (leaf, root, stem, seed and flower) from Drumstick (*M. oleifera*) tree. A total of 17,148 proteins were identified from the set of 66,079 transcripts, assembled with an N50 of 2094 bp. The expression values of 17,148 gene models were estimated by aligning this transcriptome data to the available *M. oleifera* genome [2]. Pfam [3] associations for predicted proteins were obtained for 14,624 (85.3%) proteins. Pfam domains were identified in 12,026 (∼70%) of proteins. Additionally, more than 16 thousand (∼95%) proteins found homologues in the UniProt Viridiplantae database (Table 1, Supplementary Data).

**Table 1:** Summary of transcriptome assembly and annotation.

Size of Assembly	79.79 mb
N50	2094 bp
Number of Transcripts (> 200 bp)	66,079
Average length of transcripts	1207 bp
Longest sequence	10,561 kb
Number of Proteins	17,148
Average length of proteins	433 bp
Proteins with Homology using BLAST	16,365
Homology With GO terms	15,393
Proteins with Homology using Pfam-HMM	14,624

**Fig. 1:**
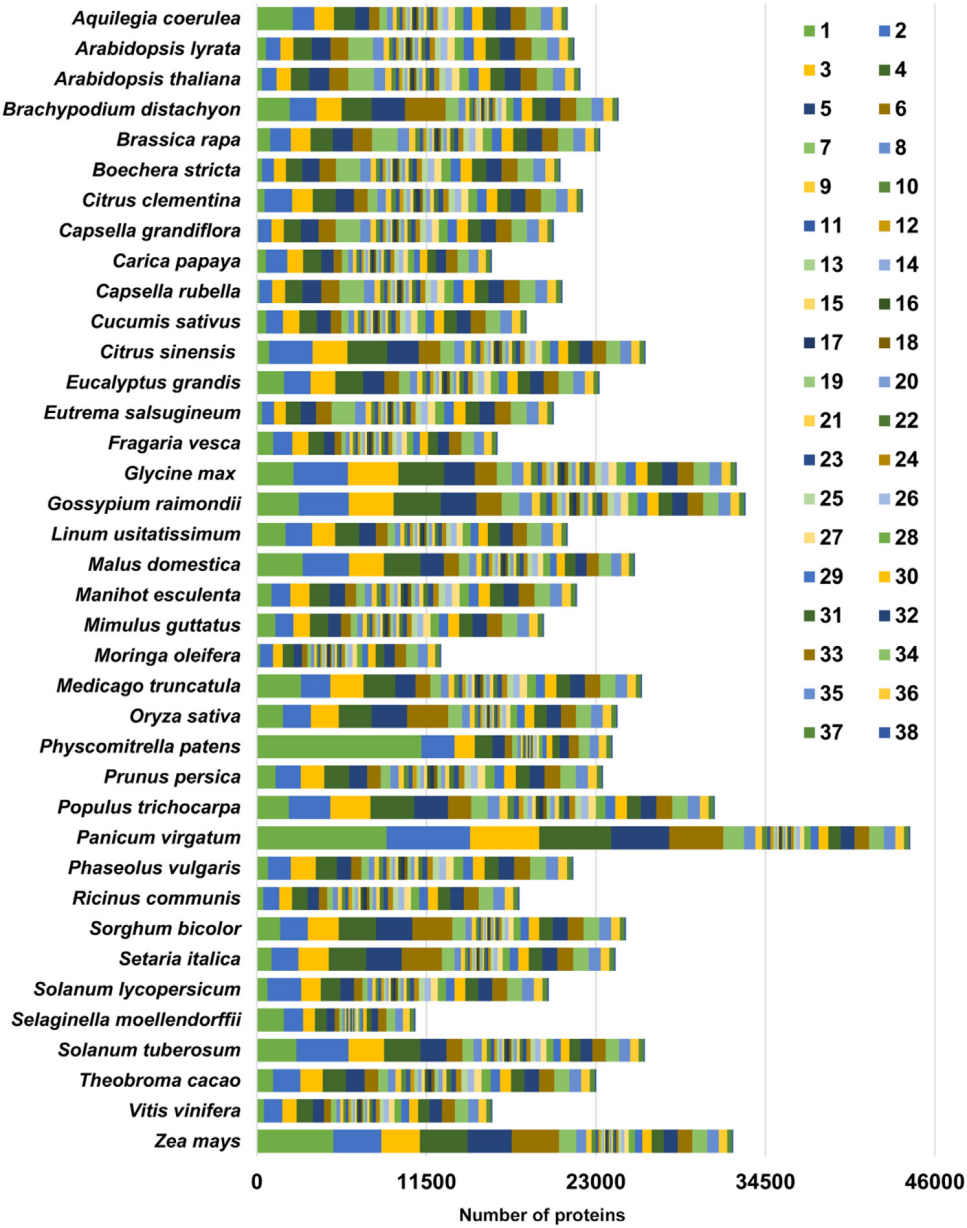
The orthogroup distribution of *M. oleifera* proteins compared across 37 plant species. The orthogroup distribution contains 38 classes from 1 to 38 – each representing the number of orthogroups from the species which are shared with zero to thirty seven of the other species in the analysis, respectively (e.g. ‘1′ - first stack of bar plot contains the unique proteins in the proteome).

**Fig. 2:**
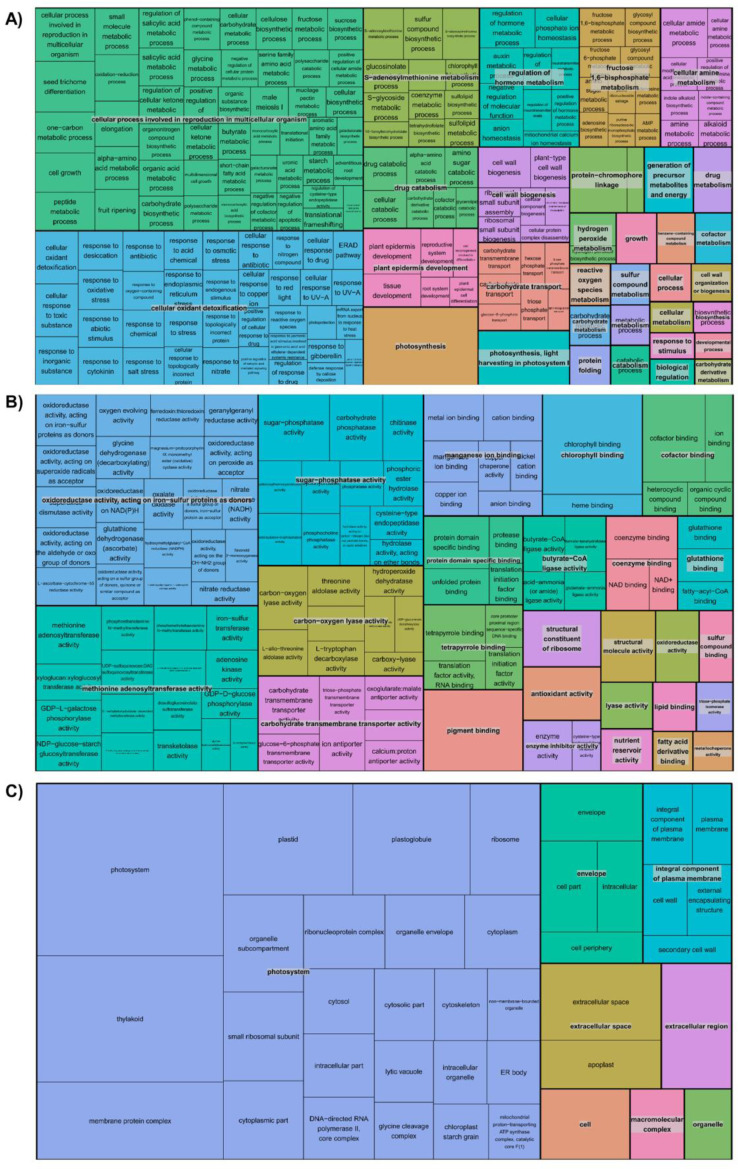
The GO terms enriched in top 100 abundant transcripts from all tissues for (A) biological process, (B) molecular function and (C) cellular component. The tree map was generated using REVIGO visualization tool. Each rectangle represent a single cluster of GO terms, visualized using different colors. Size of the each cluster indicates the p-value (less than 0.05).

**Fig. 3:**
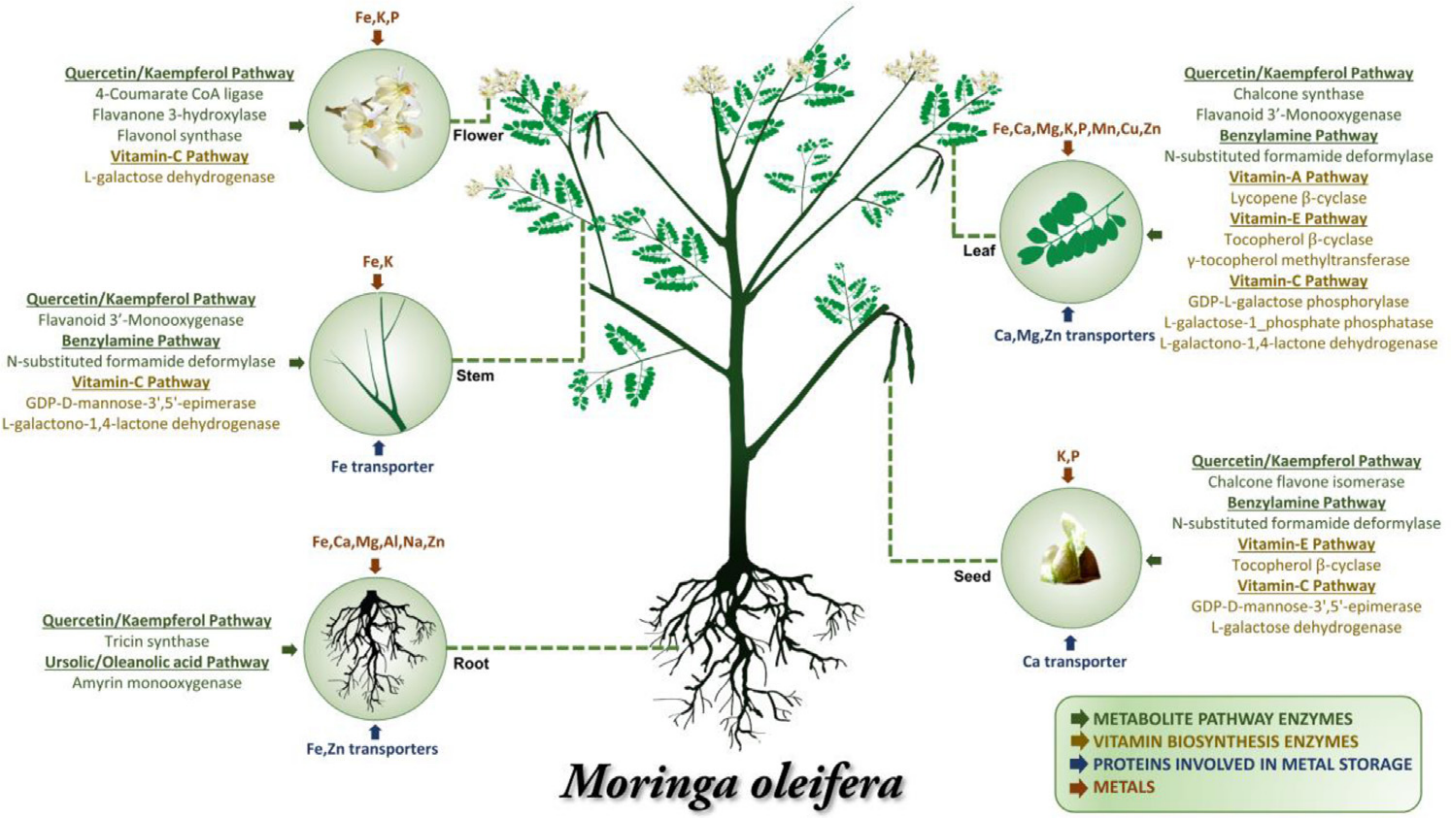
Schematic representation of tissue-specific abundance of transcripts coding for enzymes involved in secondary metabolite and vitamin biosynthesis and ion transporters. The minerals enriched in different tissues have also been indicated.

Orthology analysis was performed using two methods, OrthoMCL and ProteinOrtho. OrthoMCL analysis lead to formation of 7380 orthogroups common to selected four species. Whereas, in ProteinOrtho analysis, 102 orthogroups were observed common to all 38 species whereas 51 orthogroups were found unique to *C. papaya* and *M. oleifera* (Fig. 1, Supplementary Data). Top abundant transcripts from *M. oleifera* transcriptome were studied. Their GO terms were obtained from annotation data and enrichment analysis was performed. (Fig. 2, Supplementary Data). A set of 36 candidate genes (involved in metabolite and vitamin synthesis and ion transporters) was identified and their expression in each tissue was analysed (Fig. 3, Supplementary Data).

## 2 Experimental design, materials and methods

### 2.1 Transcriptome sequencing and assembly

RNA isolation was carried out from the five samples using Spectrum Plant total RNA kit (Sigma Aldrich), followed by treatment with Ambion-DNase1 (Thermofisher). The quality was assessed using Bioanalyzer (Agilent Technologies) and samples with RNA Integrity Number (RIN) > 7 were sequenced using Illumina HiSeq 1000, in technical duplicates (whole ten libraries). Reads were processed using Trimmomatic (v0.35) [4] and 271 million reads were retained. The assembly was guided by the reference genome [2] using Trinity (v2.4.0) [5] with default parameters.

### 2.2 Gene identification and functional annotation

Gene identification was carried out for *M. oleifera* using MAKER (v2.31.9) [6]. The gene prediction was done through Augustus using gene models from *Arabidopsis thaliana*. Pfam domains [3,7] were identified in the proteins using HMMSCAN (HMMER v3.1) with an E-value of 0.01 and Pfam library (Pfam version 31). Homologues were identified in the UniProt Viridiplantae database using BLAST (v2.7) at an E-value cutoff of 10^−3^.

### 2.3 Orthology analysis

Orthology analysis was performed on the protein coding genes identified from the transcripts of *M. oleifera* using OrthoMCL and ProteinOrtho. The OrthoMCL (v2.0.9) was implemented on *M. oleifera* and four other plant species (*Carica papaya, Theobroma cacao, Arabidopsis thaliana* and *Oryza sativa*) at an E-value cutoff of 10^−5^. The ProteinOrtho was performed using *M. oleifera* proteins and 37 other proteomes of sequenced plant genomes (as described in Pasha et al.) [1] at E-value cutoff of 10^−10^. All the proteomes were obtained from the Phytozome resource (v10.3.1) [8].

### 2.4 Differential expression of transcripts across five tissues and go term enrichment analysis

Transcriptome reads from the ten libraries derived from five tissue samples were mapped on the reference genome [2] using Tophat [9] as described in Pasha et al. [1]. Gene models were generated from each library and Fragments Per Kilobase Million (FPKM) values for each transcript were calculated using Cufflinks (v2.2.1) [10]. A merged assembly was created from the individual assemblies using cuffmerge module. Differential expression log2(fold change) of each transcript across different tissues was calculated using cuffdiff module [11].

The top 100 abundant transcripts in each tissue were examined for an enrichment of GO terms as described in Pasha et al. [1]. The Blast2GO (v5.2) [12] package was used to assign the GO terms with significance associated to a GO term based on p-value (0.05). The GO terms observed across tissues for the biological process, molecular function and cellular component were visualized using REVIGO webserver [13].

### 2.5 Proteins involved in synthesis of secondary metabolites, vitamins and transporters

A unique pipeline was developed to identify the enzymes involved in biosynthesis of medicinally important secondary metabolites and vitamins. The protein queries were identified from PlantCyc database [14] for each enzyme. These queries were aligned using Clustal Omega [15] and the alignment was used to jump-start PSI-BLAST (E-value: 10^−5^, 2 iterations) [16] for identifying *M. oleifera* protein hits. Validation of hits was performed using phylogenetic analysis and functionally important residues mapping. The abundance of the transcripts, encoding these proteins was checked across different tissues.

## Conflict of Interest

The authors declare that they have no known competing financial interests or personal relationships that could have appeared to influence the work reported in this paper.

## Appendix Supplementary materials

Image, application 1
