# Supplementary material for: Dataset for the combined transcriptome assembly of M. oleifera and functional annotation
Source: Data Brief. 2020 Mar 20;30:105416. doi: 10.1016/j.dib.2020.105416 (PMC7155000; doi:10.1016/j.dib.2020.105416)
Supplement: Supplementary file 1 [file mmc1.docx]

**Supplementary Data:** Additional data files have been provided in the link <http://caps.ncbs.res.in/download/ddat_dib/>. The index file contains details about individual files in this location.
